# Consistency of decision support software-integrated telephone triage and associated factors: a systematic review

**DOI:** 10.1186/s12911-021-01472-3

**Published:** 2021-03-21

**Authors:** Farah Islam, Marc Sabbe, Pieter Heeren, Koen Milisen

**Affiliations:** 1 Department of Public Health and Primary Care, KU Leuven, Kapucijnenvoer 35, 3000 Leuven, Belgium; 2 Department of Emergency Medicine, University Hospitals Leuven, Herestraat 49, 3000 Leuven, Belgium; 3 Department of Geriatric Medicine, University Hospitals Leuven, Herestraat 49, 3000 Leuven, Belgium; 4 Research Foundation Flanders, Egmontstraat 5, 1000 Brussels, Belgium

**Keywords:** Computerized decision support software, CDSS, Telephone triage, Unplanned care, Systematic review

## Abstract

**Background:**

In the recent decades, the use of computerized decision support software (CDSS)-integrated telephone triage (TT) has become an important tool for managing rising healthcare demands and overcrowding in the emergency department. Though these services have generally been shown to be effective, large gaps in the literature exist with regards to the overall quality of these systems. In the current systematic review, we aim to document the consistency of decisions that are generated in CDSS-integrated TT. Furthermore, we also seek to map those factors in the literature that have been identified to have an impact on the consistency of generated triage decisions.

**Methods:**

As part of the TRANS-SENIOR international training and research network, a systematic review of the literature was conducted in November 2019. PubMed, Web of Science, CENTRAL, and the CINAHL database were searched. Quantitative articles including a CDSS component and addressing consistency of triage decisions and/or factors associated with triage decisions were eligible for inclusion in the current review. Studies exploring the use of other types of digital support systems for triage (i.e. web chat, video conferencing) were excluded. Quality appraisal of included studies were performed independently by two authors using the Methodological Index for Non-Randomized Studies.

**Results:**

From a total of 1551 records that were identified, 39 full-texts were assessed for eligibility and seven studies were included in the review. All of the studies (n = 7) identified as part of our search were observational and were based on nurse-led telephone triage. Scientific efforts investigating our first aim was very limited. In total, two articles were found to investigate the consistency of decisions that are generated in CDSS-integrated TT. Research efforts were targeted largely towards the second aim of our study—all of the included articles reported factors related to the operator- (n = 6), patient- (n = 1), and/or CDSS-integrated (n = 2) characteristics to have an influence on the consistency of CDSS-integrated TT decisions.

**Conclusion:**

To date, some efforts have been made to better understand how the use of CDSS-integrated TT systems may vary across settings. In general, however, the evidence-base surrounding this field of literature is largely inconclusive. Further evaluations must be prompted to better understand this area of research.

***Protocol registration*:**

The protocol for this study is registered in the PROSPERO database (registration number: CRD42020146323).

**Supplementary Information:**

The online version contains supplementary material available at 10.1186/s12911-021-01472-3.

## Background

In the recent decades, telephone triage (TT) has become recognized as a promising tool for addressing rising healthcare demands and overcrowding in the emergency department [1–3]. TT can be defined as a process by which patients seeking unplanned health care can reach a telephone operator, which typically consists of a physician, a nurse, or a trained lay-operator for medical direction [4]. These operators are trained to evaluate patient symptoms over the phone, estimate level of urgency, and dispatch the patient to receive the most appropriate care for the presented situation (such as sending an ambulance, referring the patient to the emergency department, providing a home visit from a primary care physician, recommending self care, etc.) [4–7]. Although it has been shown that TT services are generally effective, mixed findings have been reported with regards to the safety of these systems [1–4, 8, 9]. High quality methodologies investigating the cost-related aspects are also currently lacking [1–3, 8, 10]. It is therefore generally agreed that room for improvement exists with regarding to better understanding and improving safety and costs related to CDSS-integrated TT [1–4, 8–10].

An innovative technique for advancing the quality of TT systems is the use of computerized decision support software (CDSS), which are systems that are specifically designed to support operators in the handling and managing of patient calls. Evidence suggests that the application of TT including CDSS may reduce general practitioner (GP) workload, the number of medical errors, and as well as unnecessary hospital costs [6, 11–13]. The main benefit of CDSS-integrated TT is that it seeks to standardize the decision-making process of operators to deliver high quality triage services that traditionally may have been difficult to achieve without the consultation and approval of a physician [1, 13, 14]. One underlying principle of using CDSS is that same the (i.e. consistent) triage decision is reached irrespective of the operators’ qualifications and/or prior experience [13]. With the central aim of TT services seeking to achieve these high-quality standards, the adoption of CDSS-integrated TT has been rapidly escalating internationally across different settings [1].

Despite its large potential for success, the problem with CDSS-integrated TT is that the application and practical use of these methods are complex and may vary significantly from one setting to another. Many types of CDSS-integrated TT systems are available and a wide range of possibilities exist with regards to their inherent structure, where they are used, and how they are used by operators with different professional backgrounds [12]. For instance, within the largely known National Health Services (NHS) in the UK (see 111.nhs.uk for more information) several types of CDSS are used by operators. Scholars working directly with these systems have explained that some CDSS used, such as “Access” and “Centramax,” are more prescriptive in nature—meaning that they work with predetermined types of algorithms that readily indicate the triage outcome for the operator [15]. Other types of CDSS, such as “TAS (Plain Software),” are intended to be used as interpretive software which allows the operator to “decide from available options the triage outcome they will recommend to the caller” [15]. Generally, scholars agree that in applied nurse-led TT settings, CDSS should be merely used for supporting the clinical decision-making process of the operator rather than imposing conclusive outcomes—implying that operators should always exercise their own professional knowledge in formulating a triage decision with the possibility to over-ride triage decisions that may be generated by the CDSS [2, 3, 15].

To date, there is a lack of evidence summarizing the overall quality of CDSS-integrated TT, and scientific efforts describing the efficiency and effectiveness of TT services from a larger perspective are required [4, 15]. In efforts to address the current gap in the literature, the primary aim of this systematic review is to document the consistency of decisions that are generated in CDSS-integrated TT. Furthermore, we also seek to map those factors in the literature that have been identified to have an impact on the consistency of generated triage decisions.

## Methods

The present systematic review was conducted and was reported according to the Preferred Reporting Items for Systematic Reviews and Meta-Analyses (PRISMA) Checklist [16] (see Additional file 1). The protocol for this study is also registered in the PROSPERO database (registration number: CRD42020146323).

### Search strategy

A literature search was performed in November 2019 using PubMed, Web of Science, Cochrane Central Register of Controlled Trials (CENTRAL), and the Cumulative Index to Nursing and Allied Health Literature (CINAHL) database. With the help of a librarian specialized in biomedical sciences, an exhausted list of MeSH terms and keywords related to “telephone triage” and “computerized decision support systems” were searched on PubMed. These terms were then adapted for use with other bibliographic databases in subsequent searches. The complete search strategy can be found in Additional file 2. Reference lists of all pertinent publications were also reviewed to identify any additional studies that may be relevant.

### Study inclusion and selection criteria

Articles that included a CDSS component in their TT methods and addressed either consistency of triage decision between operators and/or factors associated with triage decision were eligible for inclusion in the current study. Specifically, studies comparing agreement or disagreement of a triage outcome for a presented situation were included for analysis. Studies using a qualitative research methodology and those articles exploring the use of other types of digital support systems for triage (i.e. telehealth, wearables, etc.) were excluded.

Two authors (FI and PH) independently screened titles and abstracts to select those articles that meet the inclusion criteria. Each manuscript judged to be relevant by at least one reviewer proceeded to full text review. Full text articles were then reviewed by these authors and those judged to be irrelevant were further excluded from the review.

### Data extraction and quality assessment

Data was extracted by one author (FI) using a standardized excel database. Information regarding reference details (first author, year, country of research), main objectives of study, triage disposition categories, methods (setting, design, total number of cases to be assessed), characteristics (operator, CDSS, patient), and study results (details regarding findings, significance) were extracted. A second author (PH) reviewed accuracy of data extraction.

The quality of included studies were independently rated by two authors (FI and MS) using the methodological index for non‐randomized studies (MINORS) [17]. This is a validated instrument in which, studies are scored on an eight-item scale (for a maximum of 16 points) for non-comparative studies, or on a twelve-item scale (for a maximum of 24 points) in comparative studies. Each item is scored according to the following criteria: 0 (not reported), 1 (reported but inadequate) or 2 (reported and adequate). In the current study, an additional item was added to score the description of the CDSS software that was used. Consistent with the MINORS scoring criteria, description of CDSS was judged according to a detailed explanation of the CDSS used (i.e., such as tool name, type, description of methods, etc.). In line with scoring methods described by previous researchers [18, 19], studies were considered to have a low risk of bias if more than half the criteria were fulfilled. Specifically, a high risk of bias was considered when articles obtained a score of 0–9 (non-comparative studies) or 0–13 (comparative studies), and a low risk of bias was considered when articles obtained a score of 10–18 (non-comparative studies) or 14–26 (comparative studies).

All discrepancies regarding study inclusion, study selection, data extraction and/or quality assessment were resolved through discussion. In the case that a consensus could not be achieved, an additional author (MS, PH or KM) was consulted to make a final decision.

## Results

### Search results

**Fig. 1:**
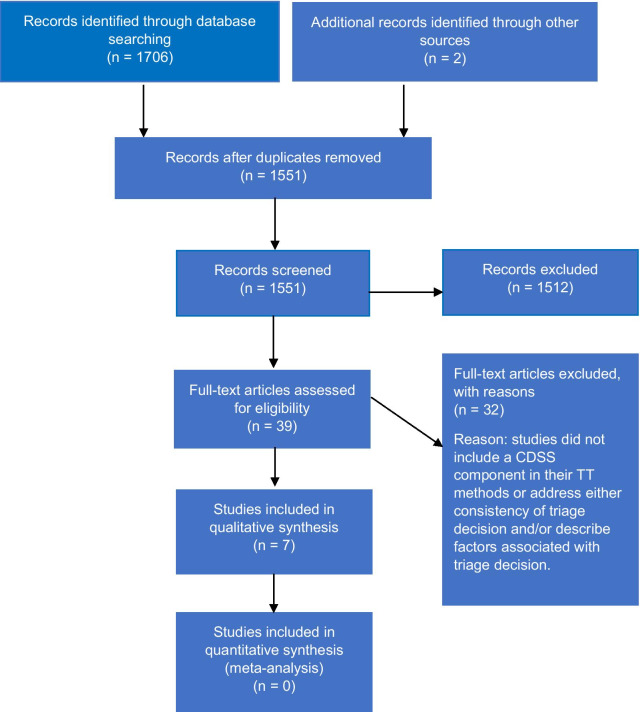
PRISMA 2009 flow diagram

A flow diagram of the study selection process is presented in Fig. 1. A total of 139 articles were identified during the PubMed search. Furthermore, 1174 articles were retrieved on Embase, 128 on Web of Science, 59 using CINAHL, 206 using the Cochrane Central database, and 2 from other sources. All duplicates were removed. A total of 1551 article were excluded from the review after screening of title and abstract due to lack of relevance. Of the 39 full-text articles that were eligible for assessment, a total of seven [13–15, 20–23] were retained for data extraction and were included the final narrative synthesis. Pooling of data could not be performed due to the small sample size and heterogeneity of study designs.

### Study characteristics

The included manuscripts were published between 1998 and 2019. Five of these articles were conducted in the UK [13–15, 22, 23], one in Belgium [21], and one in the US [20]. All of the included studies had an observational design and were based on nurse-led TT. Two of these articles [13, 20] focused specifically on pediatric-related calls while the remainder of the studies included all populations seeking unplanned care through TT. Table 1 presents a summary of the study characteristics. Complete details are available in Additional file 3.

**Table 1:** Summary of study characteristics

Reference	Country	Study design	Sample size	Operator details	Flexibility of decision	CDSS details	Patient details
Belman [20]	United States of America	Cross sectional with prospective data collection	n = 210 calls	n = 15 pediatric nurses with over 6mts of triageur experience	Yes	AHTCP	Pediatrics
Dale [14]	United Kingdom	Cross sectional, retrospective	n = 10 188 calls	n = 25 nurses experienced in community nursing and/or general practice	Not specified	TAS	Not specified
O’Cathain [15]	United Kingdom	Cross sectional	n = 119 calls	n = 1 NHS Direct nurse per call center, A&E experienced nurses with 3 months of experience with software	Yes (TAS); No (Access, Centramax); Not specified (Personal Health Adviser, AXA Assistance)	TAS; Personal Health Adviser (McKesson HBOC); Centramax (McKesson HBOC); AXA Assistance	Not specified
Varley [23]	United Kingdom	Cross sectional	n = 4474 calls	Practice nurses and nurse practitioners (n = 45)	Yes	Plain Healthcare	Not specified
Brasseur [21]	Belgium	Prospective, longitudinal study	n = 2600 calls	n = 10 nurses specialized in emergency care nurses with at least 2 years of prior experience	Yes	SALOMON algorithm	Not specified
O’Cathain [22]	United Kingdom	Cross sectional	n = 60 794 calls	n = 296 nurses	Not specified	AXA Assistance; Centramax; TAS; Access software	Not specified
Monaghan [13]	United Kingdom	Observational	n = 1281 calls	n = 22 Registered Sick Children Nurses (RSCNs) and Registered Nurses (RNs)	Not specified	TAS^2^	Pediatrics

*AHTCP* Pediatric Triage and Advice System’s computer-based telephone triage algorithms, *TAS *Plain Software telephone advice system

Scientific efforts investigating our first aim was very limited. Specifically, two of the seven articles were found to investigate the consistency of decisions that are generated in CDSS-integrated TT [15, 21]. Largely, research efforts were targeted towards the second aim of our study — all of the included articles documented factors related to operator-, patient, and/or CDSS-integrated characteristics to have an impact on the consistency of generated triage decisions. Data are summarized in Table 2.

**Table 2:** Factors related to operator-, patient, and/or CDSS-integrated characteristics impacting consistency of TT decisions

	Included articles						
	Belman [20]	Brasseur [21]	Dale [14]	Monaghan [13]	O’Cathain [15]	O’Cathain [22]	Varley [23]
Compared CDSS consistency	–	Yes	–	–	Yes	–	–
Other factors identified	Yes	Yes	Yes	Yes	Yes	Yes	Yes
Operator	Yes	–	Yes	Yes	Yes	Yes	Yes
Length of experience	–	–	–	–	–	x	–
Type of experience	x	–	x	x	x	x	–
Flexibility of decision^a^	x	x	–	–	–	–	x
CDSS	–	Yes	–	–	Yes	–	–
Inter–system consistency	x	–	–	–	–	–	–
Effectiveness^b^	–	x	–	–	x	–	–
Patient	–	–	Yes	–	–	–	–
Age of patient	–	–	x	–	–	–	-

*TT* telephone triage, *CDSS* computerized decision support software

^a^Prioritization of nurse-operator’s professional knowledge; option to overrule CDSS triage decision

^b^Validity of CDSS

Based on our ratings, the risk of bias (ROB) for studies was found to be low for all included studies [13–15, 20–23] (see Additional file 4). It is important to note that for the added item evaluating the “description of CDSS”, all studies obtained a score of one; implying that generally, descriptions of CDSS used were inadequately reported across studies.

### Consistency of TT decisions made using CDSS

In a prospective longitudinal study, Brasseur et al. [21] investigated the consistency of TT decisions using a new French-language CDSS (SALOMON) used to triage out-of-hours primary care calls in Belgium. In their research, data was collected at two time points; immediately after nurse-operators received training on how to use the CDSS (i.e. baseline), as well as 3- to 6-months later (i.e. follow-up) to determine differences in triage quality. Correlations between nurse-operators’ CDSS protocol choice (from a total of 53 protocols options that are available within SALOMON), decision regarding estimated level of triage urgency, and the gold standard (which was defined by a team of medical expert) were measured. Findings demonstrated that, nurse-operators’ CDSS protocol choice matched the gold standard 94.1% agreement at baseline and 98.7% at follow-up. Decision regarding estimated level of triage urgency between operator and the gold standard was also found to match in 93.4% and 98.5% of cases, respectively. Based on these results, authors concluded the “SALOMON” CDSS to be a very safe system for triaging patients seeking out-of-hours primary care through TT in Belgium.

In a cross-sectional analysis, O’Cathain et al. [15] examined and compared the consistency of TT decisions by nurses using four types of CDSS at four different NHS Direct call-centers including the “TAS” (Plain Software), “Personal Health Adviser” (McKesson HBOC), “Centramax” (McKesson HBOC), or “AXA Assistance” (NHS Clinical Assessment System). One-hundred nineteen low-urgency call scenarios were presented by a researcher to four different NHS Direct nurses and operators. These persons were asked to manage the call scenarios using the CDSS available at their respective call center. Results found “fair” consistency between decisions, indicating that there were significant differences in the overall agreement between nurses managing the same calls using the four different types of CDSS. Specifically, authors documented that the proportion of scenarios dispatched to accident and emergency (A&E) using the different CDSS varied between 22 and 44% while the proportion that was triaged to self care was between 9 and 29%. Overall, TT decisions made using different CDSS were not found to be consistent.

### Factors associated with consistency of TT decisions

Several factors were found to have an influence on the consistency of triage decisions generated in CDSS-integrated TT. Specifically, six of the seven studies found operator-related characteristics to have a significant influence on the outcome [13–15, 20, 22, 23]. Two of these studies also identified CDSS-type as a key element [15, 20, 21], and one described patient-related characteristics as an important factor [14].

#### Operator-related characteristics

##### Length of experience

Associations between operator-related characteristics and TT decisions were studied by several authors. In a study by O’Cathain et al. [22], it was found that nurses’ length of clinical experience was positively associated with their triage decision to refer patients to “self care”. Specifically, it was documented that nurses who had more than 20 years clinical experience were significantly more likely to triage calls to “self-care” compared to nurses with less than ten years of experience (OR = 1.41; 95% CI 1.13, 1.78).

##### Type of experience

In another article, type of experience was also found to be an important factor. Monaghan et al. [13] compared TT by registered nurses versus those who had undergone additional pediatric nursing education. Findings showed that calls concerning sick children were managed almost two minutes quicker through TT when the case was assessed by a nurse with pediatric education compared to a registered nurse. Specialized nurses were also found to resolve calls related to non-urgent medical concerns almost twice as often without further referral, meaning that they had a lower likelihood of referral to non-urgent GP services for calls related to common complaints presented by sick children.

In three studies, the consistency of TT decisions made by nurse-operators with different backgrounds was also assessed. Results showed that between operators, the consistency of triage decisions for referring patients presenting with the same or similar case was low to moderate [14, 15, 20]. Specific differences between nurses at individual call centers were not described. What is known is that in one study, authors specified that calls were handled by pediatric nurses [20]. The second study mentioned the inclusion of nurses with experience in community/general practice with training on how to use CDSS [14]. All nurses included in the third study had previously worked in the accident and emergency (A&E) department prior to their training experience in TT at the NHS Direct [15].

##### Flexibility of decisions

Furthermore, three articles [20, 21] also indicated that use of protocols for CDSS in nurse-led triage were not standardized. Though it was not specifically investigated as a direct outcome, it was indicated in these three studies that call-center operators had the flexibility to choose which CDSS algorithms they want to use. With the intention being that CDSS should only be used to support nurses in their decision-making rather than as absolute rules, operators were also allowed to overrule the TT decision that was generated by the CDSS as necessary [20, 21, 23].

#### CDSS-related characteristics

Two of the included studies also looked at correlations between CDSS-related characteristics and TT decisions. Though the findings of Brasseur et al. [21] have been described extensively in the previous sections, the study by O’Cathain et al. [15], also found that CDSS validity had an impact of TT decision. Specifically, authors also found that those CDSS with higher levels of sensitivity (such as “TAS (Plain Software)” and “Centramax”) had lower specificity, meaning that those systems which were more likely to correctly triage necessary attendances to the A&E department also had a greater likelihood of triaging unnecessary calls to the A&E department compared to the systems with low sensitivity (such as “Personal Health Adviser” and “AXA Assistance”) which were less likely to correctly triage necessary attendances to the A&E department. Findings related to the validity of the tool were not tested formally.

#### Patient-related characteristics

Only one of the included articles directly assessed patient-related characteristics (e.g. age and symptoms) that may be associated with CDSS-supported TT decision as part of the overall study objective. Dale et al. [14] showed that older persons (over 60) seeking unplanned care through TT were almost five times as likely to receive a home visit compared to patients between the ages of one and 30 years. Age was also confounded with certain key symptoms, such as “difficulty in breathing,” which was presented by one-third of patients that were over the age of 60 (compared to 9.5% of those aged less than 60 years) and also associated with higher rates of referral for a home visit compared to complaints of diarrhoea, fever or sore throat [14].

## Discussion

### Summary of findings

The objective of our systematic review was to collate the available research on CDSS-integrated TT. Despite the growing use of these services, we found that the scientific evidence addressing this topic is largely limited. A small body of inconsistent research was found to address the consistency of CDSS-integrated TT decisions [21, 22]. In one article, Brasseur et al. [15, 21] documented that the use of their CDSS was safe. In contrast, the study by O’Cathain et al. found [13] found that the decisions made by nurse-operators using CDSS-integrated TT were highly inconsistent, meaning that triage decision was significantly different across operators. There were also no standardized definitions used to describe the consistency of triage—making it impossible to draw valid scientific conclusions.

Most of the available evidence-base addressed our second research objective which aimed to investigate key factors that influence the consistency of TT decisions [13–15, 20–23]. It has been clearly documented that specific characteristics related to the operator- (such as their background, length of experience, etc.), the CDSS- (type of tool, validity), and/or the patient (older age, level of risk, other biases, etc.) may have a significant influence on the operators’ estimated level of triage urgency and ultimately, final TT decision outcome. Though these factors are important, it is difficult to draw complete conclusions regarding CDSS-integrated TT decisions without addressing the larger effects of organizational and environmental forces which may also directly and indirectly drive final decision outcomes.

**Fig. 2:**
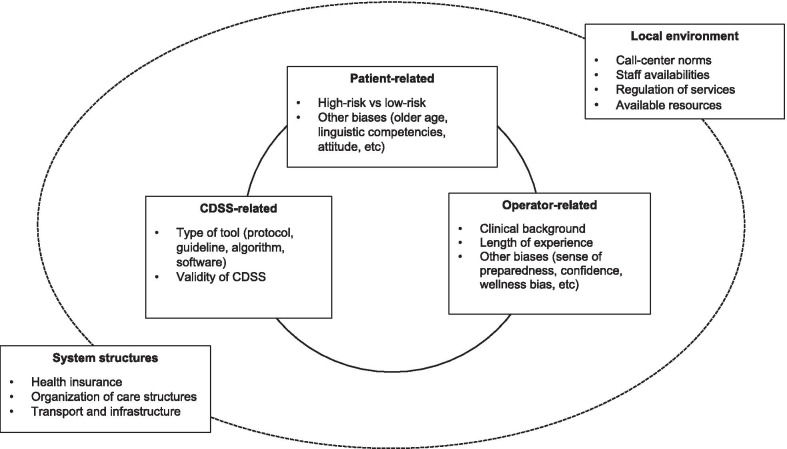
Factors associated with estimated level of triage urgency

Specifically, we argue that two key external domains associated with estimating level of triage urgency include system structures (i.e. health insurance, organization of care structures, transport and infrastructure, etc.) and the local environment (i.e. call-center norms, staff availabilities, regulation of services, available resources, etc.) (Fig. 2). To better understand the implication of these factors, we can think about the capacities and resources of TT call-centers to manage incoming calls. Despite the use of standardized CDSS systems, call-center norms may vary according to acceptable levels of over- and under- triage for managing complex cases in relation to the accessibility of medical staff available on site. For instance, in TT centers where a nurse or GP may not be readily present for further consultation related to a call, lay operators may be prompted to scale up the urgency of a case despite CDSS recommendations to prioritize patient safety. In another example, we can consider the call of a vulnerable older patient without access to private transportation for a hospital visit (i.e. with no possibility to drive or obtain assistance from a friend or family). In such cases, initial CDSS recommendations may be overruled for the dispatch of an emergency vehicle to manage transport and infrastructure-related barriers and to provide assistance to the patient. To our knowledge, very few research efforts have been made to date which recognize and clearly address the impact of these components using rigorous scientific methods.

### Limitations

A fairly low number of studies were included in the current review. Though this is largely related to the scare evidence base surrounding this topic, a broader research question using less stringent study inclusion/exclusion criteria may have illuminated a larger scope of relevant findings within the existing literature.

Nonetheless, our review has highlighted some important discoveries in the field of CDSS-integrated TT. To date, only observational research efforts based on nurse-led TT have been prompted. The methodological quality of the included articles were found to have a low risk of bias (see Additional file 4). However, as none would adhere to the standards used by the Cochrane Effective Practice and Organisation of Care Review Group (EPOC), the overall evidence base on this topic is probably of low quality [24]. In general, insufficient information was identified by scholars with regards to the descriptions of the CDSS that were used. Furthermore, it was largely unclear whether evaluation of study objectives were blinded. Ambiguities were also found with regards to information on prospective power analyses of studies. These limitations make it difficult to draw any inferences based on the associations found in the literature.

Furthermore, our evidence cannot be extended to make inferences about operators with different levels of medical experience and training. Knowing that TT services are often also led by lay-operators with non-medical backgrounds, scientific studies conducted in this field need to further investigate these differences [4]. Finally, the evidence base surrounding this topic is extremely scarce and we know very little about the content and procedure of different CDSS that are used in the literature. Without additional information addressing these barriers, the overall quality of CDSS cannot be adequately compared. Assessment of these associations with a large enough sample size and different types of CDSS is necessary to make conclusive scientific judgements.

### Challenges of reporting on quality of CDSS

A key challenge we faced while conducting this study is that the information available regarding the different types of CDSS that are used in the literature are insufficient to make scientific comparisons. Comprehensive details describing different types of CDSS that are used and the standards/norms regarding how they are used across settings are rarely reported in manuscripts. In efforts to address this issue, we attempted to obtain further information regarding the CDSS that were widely used across studies included in this research paper. Online searches were carried out and some authors/services were contacted to obtain additional information regarding those CDSS that were reported. However, our success was rather limited. A rigorous investigation of CDSS quality was therefore not possible for the purposes of the current manuscript.

### Future research considerations

As a result of the COVID-19 pandemic, the role of TT has surged quicker than ever to play a critical role in effectively managing patients. As we rapidly shift towards a society in which remote-based triage technologies are at the frontline of patient management, it is imperative to reflect on the highest standards of triage that we can achieve using these emerging systems. At the same time, it is important to remember that TT is not physical triage. Therefore, while prompt systematic efforts to comprehensively assess and evaluate the potential of these tools is necessary, we must also consider the intrinsic limitations of these tools. Until recently, a large limit of TT has been the ability to record physical patient measurements. However, with the introduction of “telehealth” services and non-invasive “wearables” including sensing systems capable of remotely monitoring the major vital signs of a patient (i.e. body temperature, pulse rate, respiration rate, blood pressure, etc.), these possibilities continue to expand. Guidelines created by experts should be adapted to reflect the rapidly evolving capacities of TT techniques.

From a scientific point of view, substantial efforts are required to further establish how remote- triage technologies can be used to successfully manage healthcare demands in the population. At the same time, the current pandemic situation has accelerated the growth and use of these systems exponentially, and it is imperative that research efforts catch up. Fruitful research endeavors should therefore seek to further identify and synthesise findings of widely used TT methods, better understand how different factors may influence CDSS-integrated TT (as well as other remote-based triage) using established criteria such as the GUIDES checklist [25], and clearly document how these systems are used in practice.

## Conclusion

CDSS-integrated TT has become a prominent instrument for managing unplanned care needs in the population. Our systematic review demonstrates that some efforts have been made to better understand how the use of these systems may vary across settings. In general, however, the evidence-base surrounding this field of literature is to date, very limited and largely inconclusive. Further scientific investigations must be prompted to make conclusive statements about the effectiveness of these systems.

## Supplementary Information

**Additional file 1**. PRISMA Checklist.

**Additional file 2**. Search Strategy Used for Databases.

**Additional file 3**. Complete Study Characteristics.

**Additional file 4**. Quality Appraisal of Included Studies. 

## Data Availability

All data generated or analyzed during this systematic review are included in this published article and/or its additional information files.

## Abbreviations

CDSS: Computerized decision support software
TT: Telephone triage
MINORS: Methodological Index for Non-Randomized Studies
