# Supplementary material for: Consistency of decision support software-integrated telephone triage and associated factors: a systematic review
Source: BMC Med Inform Decis Mak. 2021 Mar 21;21:107. doi: 10.1186/s12911-021-01472-3 (PMC7981379; doi:10.1186/s12911-021-01472-3)
Supplement: Supplementary file 2 — Additional file 2. Search Strategy Used for Databases. [file 12911_2021_1472_MOESM2_ESM.docx]

Additional file 2: search strategy used for databases

| Database | Keywords and MeSH Terms |
| --- | --- |
| PUBMED | (“telephone triage”[Title/Abstract]  OR “hotlines” [MeSH Terms]  OR hotline* [Title/Abstract]  OR “telephone triage and advice services” [Title/Abstract]  OR advice-service* [Title/Abstract]  OR “teleconsultation” [MeSH Terms]  OR “teleconsultation” [Title/Abstract]  OR “telenursing” [MeSH Terms]  OR “telenursing” [Title/Abstract])  AND  (“decision support systems, clinical”[MeSH Terms]  OR decision-support-system* [Title/Abstract]  OR Computer-decision-support-software*[Title/Abstract]  OR “computer-assisted” [Title/Abstract]  OR “computer-supported” [Title/Abstract]  OR “algorithm*”  OR “appointments and schedules”[MeSH Terms]  OR “clinical-support-system*”[Title/Abstract]) |
| EMBASE | (‘telephone triage’/exp OR ‘telephone triage’:ti,ab,kw  OR ‘hotline’/de OR ‘hotline*’:ti,ab,kw  OR ‘advice service*’:ti,ab,kw  OR ‘teleconsultation’ OR ‘teleconsultation’:ti,ab,kw  OR ‘telenursing’/exp OR ‘telenursing’:ti,ab,kw)  AND  ('decision support system'/de OR ‘decision support system ‘:ti,ab,kw  OR ‘software’/de OR ‘software’:ti,ab,kw  OR ‘computer decision support-software’:ti,ab,kw  OR ‘computer assisted diagnosis’/exp OR ‘computer assisted’:ti,ab,kw  OR ‘computer supported’:ti,ab,kw  OR ‘algorithm’/de OR ‘algorithm’:ti,ab,kw  OR ‘patient scheduling’/exp OR ‘appointments and schedules’:ti,ab,kw  OR ‘clinical decision support system’/exp OR ‘clinical decision support system’:ti,ab,kw) |
| CINAHL | (TI “telephone triage” OR AB “telephone triage”  OR TI “hotline*” OR AB “hotline*”  OR TI “telephone triage and advice services” OR AB “telephone triage and advice services”  OR TI “advice service*” OR AB “advice service*”  OR TI “teleconsultation” OR AB “teleconsultation”)  AND  (MH “decision support systems, clinical”  OR TI “decision-support-system*” OR AB “decision-support-system*”  OR TI Computer-decision-support-software* OR AB Computer-decision-support-software*  OR TI “computer-assisted” OR AB “computer-assisted”  OR TI “computer-supported” OR AB “computer-supported”  OR TI “algorithm*” OR AB “algorithm*”  OR MH “appointments and schedules”  OR TI “clinical-support-system*” OR AB “clinical-support-system*”) |
| Cochrane Central Register of Controlled Trials (CENTRAL) | \| #1 \| [mh “hotlines”] OR [mh “teleconsultation”] OR [mh “telenursing”] \| \| --- \| --- \| \| #2 \| (telephone triage) ti,ab,kw OR (hotline) ti,ab,kw OR (telephone triage and advice services) ti,ab,kw OR (advice service) ti,ab,kw OR (teleconsultation) ti,ab,kw OR (telenursing) ti,ab,kw \| \| #3 \| [mh “decision support systems, clinical”] OR [mh “appointments and schedules”] \| \| #4 \| (decision-support-system*) ti,ab,kw OR (computer decision support software*) ti,ab,kw OR (computer assisted) ti,ab,kw OR (computer supported) ti,ab,kw OR (algorithm) ti,ab,kw OR (clinical-support-system) ti,ab,kw \| \| #5 \| (#1 or #2) and (#3 or #4) \| |
