# Supplementary material for: Consistency of decision support software-integrated telephone triage and associated factors: a systematic review
Source: BMC Med Inform Decis Mak. 2021 Mar 21;21:107. doi: 10.1186/s12911-021-01472-3 (PMC7981379; doi:10.1186/s12911-021-01472-3)
Supplement: Supplementary file 3 — Additional file 3. Complete Study Characteristics. [file 12911_2021_1472_MOESM3_ESM.docx]

| Additional file 2. Complete study characteristics (data extraction form) | | | | | |
| --- | --- | --- | --- | --- | --- |
| **Reference** | **Objective** | **Triage categories** | **Methods** | **Characteristics** | **Findings** |
| Belman [20]  US | To assess the consistency  of triage dispositions among experienced telephone  triage nurses at a children’s hospital–based call center and  To determine agreement between nurse disposition and  protocol disposition using mock parents delivering actual  cases. | - Call 911 - Come in immediately - Come within 4 hours - Call your doctor in 24 hours - Call your doctor in 72 hours - Home care instructions | **Setting:**  Private pediatrician clinics, Denver, USA  **Design:**  Cross-sectional with prospective data collection, blinded, observation  **Cases assessed:**  n= 210 calls  (aim was to have same 15 mock calls assessed by 15 nurses = 225) | **Operator:**  Pediatric Nurses with over 6mts of triageur experience (n=15)  **Flexibility in TT decision =** yes  **CDSS:** Pediatric  Triage and Advice System’s computer-based telephone  triage algorithms (AHTCP)  **Patients:** Pediatrics | **Consistency of triage decision between nurses**  k = 0.46; 95% confidence interval, 0.43–0.49 (collapsed categories)  **Sig. = Yes**  Moderate interrater reliability was found among experienced call center nurses.  **Consistency of triage decision between nurses and CDSS**  Range of agreement was found to vary between 33% to 100%.  **Sig. =** No  Few cases, limited  ability to detect a difference between nurse and protocol disposition  No differences in length of call or information elicited between cased receiving urgent and nonurgent dispositions |
| Dale [14]  UK | To describe the way that calls are assessed, triaged and advised according to patient characteristics and nurse managing the call | - Advised to call 999 - Advised to go to A&E - Home visit - Attend base - See GP routinely - Nurse advice - Referred to district nurse - Other | **Setting:**  OOH telephone triage advice service in general practice, London, UK  **Design:**  Cross sectional, retrospective  **Cases assessed:**  n= 10 188 calls  (avg of 407 calls per nurse) | **Operator:**  Nurses (n=25) experienced in community nursing and/or general practice  **Flexibility in TT decision =** not reported  **CDSS:** TAS software  **Patients:** Not specified | **Triage decision based on patient age and complaint**  Call outcome varied with age and complaint.  40.7% of those over 60 received home visit (X^2^= 866.4, df=3, p<0.0001)  Patients complaining of “difficulty in breathing” were twice as likely to be referred for a home visit compared to complaints o diarrhea, fever, or sore throat  **Sig. =** Yes  Outcome of calls varied with age. Older patients (over 60) sig more (5x more likely to receive home visit; confounded with key symptoms, such as “difficulty in breathing”  **Call outcome based on nurse characteristics**  Considerable variation. No p-values provided  **Sig. =** Not clear  Considerable variation in call outcomes between the assessing nurses |
| O’Cathain [15]  UK | To examine the consistency of triage outcomes by nurses using four types of computerized decision support software in NHS direct | - 999 ambulance - A&E department - GP immediately - (up to four hours), - GP later, - other service - self-care | **Setting:**  NHS direct, UK  **Design:**  Cross sectional  **Cases assessed:** n=119 calls | **Operator:**  NHS Direct nurse (n=1 per call center at 4 NHS centers), A&E experienced nurses and 3mths experience with software  **Flexibility in TT decision =**  Yes (TAS); No (Access, Centramax); Not reported (Personal Health Adviser, AXA  Assistance)  **CDSS:**  TAS (Plain Software)  Personal Health Adviser (McKesson  HBOC),  Centramax (McKesson HBOC).  AXA  Assistance (NHS Clinical Assessment System)  **Patients:** Not specified | **Consistency of triage decision between nurses**  Overall level of agreement between the nurses using the four systems was “fair” (k=0.375, 95% CI: 0.34 to 0.41).  **Sig. =** yes, but not clearly stated in article  Large differences in outcome between nurses using different software systems  to triage the same calls.  **CDSS validity**  CDSS with higher sensitivity had lower specificity  **Sig. =** not clear; no formal analysis conducted |
| Varley [23]  UK | To investigate whether the professional characteristics of primary care nurses (level of experience, qualifications) undertaking computer decision supported software telephone triage are related to call disposition | Follow-up  No follow-up  Triage categories not reported extensively in current paper but can be derived through categories triage categories available through the NHS system (see O’Cathain et al., 2003 for example) | **Setting:**  general practices across four  regions of England.  **Design:**  Cross sectional  **Cases assessed:** n= 4474 calls | **Operator:**  Practice nurses and nurse practitioners (n=45)  **Flexibility in TT decision =** yes  **CDSS:** Plain Healthcare  **Patients:**  Not specified | **Nurse characteristics**  Practice nurse more likely to dispose call compared to nurse practitioner; less prepared nurse more likely to dispose call  **Sig. =**Yes;  Based on the final model, including nurse practitioner status, prescriber status, and perceived preparedness (Table 3), the marginal probability for a patient being followed-up in practice was 0.90 with a 95% CI of 0.88–0.93  for practice nurses (Table 3); for nurse practitioners the probability was 0.66 (95% CI 0.51–0.81). |
| Brasseur [21]  Belgium | To evaluate the criterion validity/ effectiveness of a specific French-language triage algorithm called SALOMON | - Emergency Medical Services Intervention - Non-urgent Emergency Department Consultation - Primary Care Physician Home - Primary care Physician Delayed visit | **Setting:**  two facilities of the  University Hospital of Liege  **Design:**  Prospective, longitudinal study  **Cases assessed:**  n= 2600 calls (130 calls per nurse at two different time points) | **Operator:**  Nurses (n=10) specialized in  emergency care nurses with at least 2 years of prior experience  **Flexibility in TT decision =** yes  **CDSS:**  SALOMON algorithm  **Patients:** Not specified | **Consistency of triage decision between nurses and CDSS**  High degree of agreement between nurses and gold standard  Nurses choice for SALOMON level matched reference in 93.4% of cases at T1 and 98.5% of cases at T2 (p<0.0001)  Comparison of algorithm selected between nurse and reference revealed 94.1% agreement at T1 and 98.7% at T2 (p<0.0001)  **Sig.=**Yes  **CDSS validity**  Conclusion regarding validity derived through above findings.  The French-language triaging algorithm shows high sensitivity and specificity of the tool. Very safe system with limited mistriage. |
| O’Cathain [22]  UK | To determine whether nurses with different clinical backgrounds make different triage decisions in NHS Direct, the 24-hour telephone helpline staffed by nurses | - Emergency ambulance - A&E department, - GP immediately - GP later - Self-care - Other service   (categories for analysis were dived between not self-care and self-care) | **Setting:**  NHS Direct sites  **Design:**  Cross-sectional  **Cases assessed:**  n= 60 794 calls | **Operator:**  Nurses (n=296)  **Flexibility in TT decision =** not reported (see O’Cathain et al., 2003)  **CDSS:**  NHS system  Centramax  TAS  Access software  **Patients:** Not specified | **Nurse characteristics**  Length of clinical experience (*P*= 0.016)  Type of clinical experience (*P*= 0.064)  **Sig.** = Yes  Variability was partly explained by the length and type of clinical experience of nurses. |
| Monaghan [13]  UK | To assess whether the length and end triage outcome given to callers phoning on behalf of children complaining of common symptoms were associated with nurses’ qualifications and experience | - 999 - A&E - GP now - GP routine - Nurse advice | **Setting:**  NHS Direct call centre in the West Midlands  **Design:**  Observational | **Operator:**  Nurse (Registered Sick Children Nurses (RSCNs) and  Registered Nurses (RNs)) (n=22)  **Flexibility in TT decision =** not reported  **CDSS:**  Plain Software telephone advice system (TAS) version 5.1 (n= 1281  **Patients:** Pediatrics | **Length of calls**  Median length of calls received by RNs was 9.0 minutes compared to 7.0 minutes for RSCNs (Mann-Whitney *U*=156218.5, *Z=*7.39, *P*= <0.001)  **Sig**. **=** Yes  **Triage decisions**  Total number of callers referred to GP appointment by RSCNs was significantly higher than that of RNs. Of 191 lls refers as a routine GP visit, 122 (64%) were by RSCNs while 69 were by RNs (chi-square=13.65, df=1, *P<* 0.001)  **Sig**. **=** Yes  The number of callers referred on to routine GP appointment by RSCNs was significantly higher than that of RNs.  No other significant differences were detected in call outcomes between groups |
