# Supplementary material for: Consistency of decision support software-integrated telephone triage and associated factors: a systematic review
Source: BMC Med Inform Decis Mak. 2021 Mar 21;21:107. doi: 10.1186/s12911-021-01472-3 (PMC7981379; doi:10.1186/s12911-021-01472-3)
Supplement: Supplementary file 4 — Additional file 4. Quality Appraisal of Included Studies. [file 12911_2021_1472_MOESM4_ESM.docx]

Additional file 4**.** Quality appraisal of included studies with Methodological Index for Non-Randomized Studies (MINORS)

| **Table I: Final MINORS score** | | | | | | | |
| --- | --- | --- | --- | --- | --- | --- | --- |
|  | Belman[20] | Brasseur [21s] | Dale  [14] | Monaghan [13] | O’Cathain [15] | O’Cathain [22] | Varley [23] |
| A clearly stated aim | 2 | 2 | 2 | 2 | 2 | 1 | 2 |
| Inclusion of consecutive patients | 2 | 2 | 2 | 2 | 1 | 2 | 2 |
| Prospective data collection | 2 | 2 | 2 | 2 | 1 | 2 | 2 |
| Endpoints appropriate to the aim of the study | 2 | 1 | 2 | 2 | 2 | 2 | 2 |
| Unbiased assessment of the study endpoint | 2 | 0 | 0 | 1 | 1 | 1 | 0 |
| Follow-up period appropriate to study aim | 2 | 2 | 0 | 2 | 1 | 2 | 2 |
| Loss to follow up less than 5% | 2 | 2 | 2 | 2 | 2 | 0 | 1 |
| Prospective calculation of the study size | 0 | 0 | 1 | 0 | 0 | 0 | 1 |
| An adequate control group | N/A | N/A | N/A | N/A | N/A | N/A | N/A |
| Contemporary groups | - | - | - | - | - | - | - |
| Baseline equivalence of groups | - | - | - | - | - | - | - |
| Adequate statistical analyses | - | - | - | - | - | - | - |
| Randomisation | - | - | - | - | - | - | - |
| Description of CDSS^a^ | 1 | 1 | 1 | 1 | 1 | 1 | 1 |
| Total score | 15/18 | 12/18 | 12/18 | 14/18 | 11/18 | 11/18 | 13/18 |
| Risk of bias assessed^b^ | low | low | low | low | low | low | low |
| ^a^ CDSS = computerized decision support software  ^b^ See Table II | | | | | | | |

*The items were scored 0 (not reported), 1 (reported but inadequate) or 2 (reported and adequate). The Methodological Index for Non-Randomized studies is a twelve-item instrument. In this table, a thirteenth item was added concerning the use of randomization.

| **Table II: Risk of bias criteria assessment for MINORS** | | |
| --- | --- | --- |
| **Score (non-comparative)** | **Score (comparative)** | **Risk of bias** |
| 0-9 | 0-13 | High |
| 10-18 | 14-26 | Low |

**Risk of bias assessment**

Quality of the included studies were independently rated by two authors (FI and MS) using the methodological index for non‐randomized studies [16]. This is a validated instrument in which studies are scored on an eight-item scale (for a maximum of 16 points) for non-comparative studies, or on a twelve-item scale (for a maximum of 24 points) in comparative studies. Each item is scored according to the following criteria: 0 (not reported), 1 (reported but inadequate) or 2 (reported and adequate). In the current study, and additional item was added to score the description of the CDSS software that was used. Consistent with the MINORS scoring criteria, description of CDSS was judged according to a detailed explanation of the CDSS used (i.e., such as tool name, type, description of methods, etc). In line with scoring methods described by previous researchers [17, 18], studies were considered to have a low risk of bias if more than half the criteria were fulfilled. Specifically, a high risk of bias was considered when articles obtained a score of 0-9 (non-comparative studies) or 0-13 (comparative studies), and a low risk of bias when articles obtained a score of 10-18 (non-comparative studies) or 14-26 (comparative studies). All discrepancies in ratings were resolved by discussion. In the case that a consensus could not be achieved, an additional author (PH or KM) was consulted to make a final decision. No articles were excluded from the narrative synthesis based on article quality. A meta-analysis was not possible due to heterogeneity of studies.
